# Don't Forget to Bring Your Cheque Book

**Published:** 1983

**Authors:** 


### ANNOTATIONS

**'DON'T FORGET TO BRING YOUR CHEQUE BOOK'**

Texas, the land of big hats, big cigars and even bigger medical bills. I flew 10 hours non-stop to see my week-old grandson – born with jaundice and a fractured clavicle. He and his mother had been in hospital for 3 days. Soon after my arrival the bills came. It seemed immaterial that my daughter-in-law had delivered her son by natural childbirth with no anaesthetic for there were two items to be paid for –

| | |
|---|---|
| *Total anaesthesia material* | $27.00 |
| *Anaesthesiologist* | $225.00 |

Nor that she had refused to have her son circumcised – item 5 –

| | |
|---|---|
| *Circumcision tray* | $16.50 |

There were 17 items listed on the child's bill; top of this list was for

| | |
|---|---|
| *Nursery room* | $189.00 |

then came

| | |
|---|---|
| *Treatment for jaundice* | $100.00 |

down to a

| | |
|---|---|
| *Bath kit* | |
| *3 oz tube of Vaseline* | |
| *Take home thermometer* | |
| *Total* | $377.45 |

his mother did not get off quite so lightly for there were 52 items on her bill to be accounted for – she had needed

| | |
|---|---|
| *Air ring* | $8.80 |
| *6 Senokot tablets* | $2.10 |
| *4 oz Milk of Magnesia* | $2.00 |
| *Pre-op tray* | |
| *Linen savers* | |
| *Sterile water* | |
| *Bandaids* | |
| *Total drugs* | $86.00 |

Sterile scrub suit for father – the list seemed endless. The only article free was the loan of a Polaroid camera to photograph the birth. There were still more bills for after all there had been a doctor present for a fee of – $850.00

and several ante-natal classes to be paid for. As I left Texas yet another bill plopped through the letter box – of course I forgot that we had attended two post-natal clinics – $50.00

| | |
|---|---|
| *Grand total* | $2740.85 |

During my visit each dollar was worth £0.58 and the sterling cost was £1589.69.

I was invited to a party before coming home and my American companion told me she was due to go in to hospital for a small operation. She had rung the hospital to find out if there was anything particular she needed to take in – 'Nothing much' she was told, 'but don't forget to bring your cheque book. Have a nice day.'

J.M.P. (*Chartered Physiotherapist*)

